# Lack of physiotherapy resources restricts exercise prescription for patients with chronic kidney disease—the EUropean SUrvey on REnal EXercise (EUSUREX)

**DOI:** 10.1093/ckj/sfaf291

**Published:** 2025-09-17

**Authors:** Naomi Clyne, Adamasco Cupisti, Clemens Grupp, Evangelia Kouidi, Eva Segura-Ortí, Pasquale Fabio Provenzano, Vicent Esteve-Simó, Giovanni Tripepi, Carmine Zoccali, the EUSUREX Working Group, David Plate, Horst Berger, Andreas Fürsch, Thomas Döltz, Norgit Meyer, Cosima Pohle, Dominik Kahle, Michael Schömig, Pawlos Ichtiaris, Roman Günthner, Gunnar Bücker, Bernadette Assenbrunner, Kerstin Fiegler, Vasiliki Michou, Ourania Kougioumtzidou, Zagorianakos Antonios, Kakaletri Konstantina, Liakopoulos Vasileios, Maurizio Gallieni, Manuel Ferraro, Elena Mancini, Stefano Santarelli, Antonio Selvi, Paolo Monardo, Giovanni Campolongo, Donatella Andreoli, Annalisa Carta, Shaira Martínez Vaquera, Karl Bjurström, Lars Ekholm, Noomi Lundgren

**Affiliations:** Lund University, Skåne University Hospital, Faculty of Medicine, Department of Clinical Sciences Lund, Nephrology, Lund, Sweden; University of Pisa, Department of Clinical and Experimental Medicine, Pisa, Italy; Georg-August University, Faculty of Medicine, Department of Nephrology and Rheumatology, Göttingen, Germany; Laboratory of Sports Medicine, Department of Physical Education and Sport Science, Aristotle University of Thessaloniki, Thessaloniki, Greece; Universidad Cardenal Herrera-CEU, CEU Universities, Department of Physiotherapy, Valencia, Spain; National Research Council, Institute of Clinical Physiology, Reggio Calabria, Italy; Consorci Sanitari de Terrassa, Spain; National Research Council, Institute of Clinical Physiology, Reggio Calabria, Italy; Renal Research Institute, New York, NY, USA Institute of Molecular Biology and Genetics (Biogem), Ariano Irpino, Italy Associazione Ipertensione Nefrologia Trapianto (IPNET), c/o Nefrologia, Grande Ospedale Metropolitano, Reggio Calabria, Italy

**Keywords:** chronic kidney disease, exercise, healthcare system, physiotherapy, survey

## Abstract

**Background:**

We hypothesized that the main barriers to integrating exercise in routine care of patients with chronic kidney disease (CKD) were structural within European healthcare systems rather than due to resistance among healthcare professionals or patients.

**Materials and methods:**

This descriptive cross-sectional study used anonymized questionnaires to investigate nurses’ and physicians’ attitudes and practice towards exercise in patients with CKD, structural support within the healthcare system, and patients’ experience of exercise prescriptions. Kidney units were randomly selected in Germany, Greece, Italy, Spain, and Sweden.

**Results:**

In total, 352 (35% male) nurses; 143 (54% male) physicians participated; 96% and 98%, respectively, believed exercise was beneficial. Of them, 59% (CI 50%–67%) prescribed exercise ‘always or often’ (*I*^2^ 91%), ranging from 15% in Germany to 86% in Sweden. Of the facilities, 48% (CI 39%–56%) did not have a physiotherapy unit (*I*^2^ 98%); six% (CI 3%–12%) did: in Italy one% (CI 0.03%–7%); in Sweden 86% (CI 42%–99%), (*I*^2^ 97%). Seven% (CI 3%–12%) had physiotherapists and exercise programmes; eight% (CI 4%–13%) for all treatment modalities: in Italy four% (CI 1%–10%) and five% (CI 1%–12%), respectively; in Sweden 71% (CI 29%–96%) and 86% (CI 42%–99%), (*I*^2^ 87% and 97%, respectively). Only Sweden reimbursed physiotherapy costs for all patients. In total, 1235 patients participated: those with CKD 4–5 (*n* = 137, male 62%); peritoneal dialysis (*n* = 40, male 60%); home haemodialysis (*n* = 30, male 63%); institutional haemodialysis (*n* = 928, male 59%); and kidney transplant (*n* = 100, male 62%). Between 9% and 37% of all patients reported having received an exercise programme, among those one- to two-thirds had continued to exercise.

**Conclusions:**

Physicians and nurses regarded exercise as important for patients with CKD. Physical performance was not assessed. Most patients had not received exercise prescriptions. All countries, except Sweden, lacked physiotherapy resources and reimbursement strategies. Healthcare systems need to recognize the beneficial effects of exercise and provide adequate resources.

## Graphical Abstract

**Graphical Abstract:**
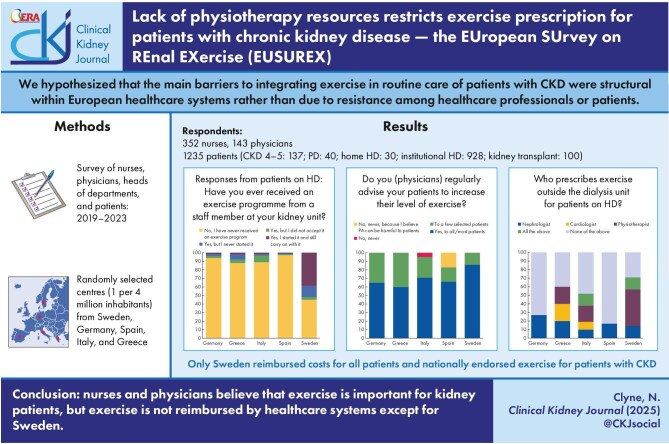

KEY LEARNING POINTS **What was known**:

- Nurses and physicians believed that exercise could be harmful for patients with chronic kidney disease (CKD) and on kidney replacement therapy (KRT).
- They believed that patients were too tired, too sick, or just uninterested in exercise.
- Nurses and physicians felt that they lacked knowledge, training, and time to prescribe exercise to their patients.

**This study adds**:

- This study shows a switch in attitudes among nurses and physicians in Western Europe, who overwhelmingly believe that exercise is beneficial for patients with CKD and on KRT. There is a large variation between countries in physicians’ actual recommendations to exercise.
- All countries except Sweden lacked dedicated physiotherapists to assess physical performance and prescribe exercise. Despite healthcare professionals’ positive attitude towards exercise most patients reported that they had not received exercise programmes.
- All countries except Sweden lacked resources to prescribe and implement exercise due to lack of physiotherapy resources and reimbursement policies, which did not pay for physiotherapy for patients with CKD and on KRT.

**Potential impact**:

- This study highlights the need for healthcare systems to reimburse costs for physiotherapist led exercise in patients with CKD and on KRT.

## INTRODUCTION

Observational studies have shown significant associations between decreased mortality and morbidity and level of physical activity in patients with chronic kidney disease (CKD) [1, 2]. Some large, long-term randomized controlled trials (RCT) have shown that exercise increases physical performance in patients with CKD, including the frail and multimorbid [3–6]. Moreover, lower rates of hospitalization were reported in patients who participated in exercise [4, 6].

Earlier studies have shown that both physicians and nurses believed that patients with CKD are too sick, too tired, or just lack motivation to exercise [7–9]. Interestingly, one study showed that physicians who were physically active themselves were more prone to prescribe exercise compared with sedentary physicians [10]. Recent studies have shown that interest, motivation, and adherence were high both in patients on dialysis and with CKD 3–5 [3–6]. However, an important factor for success is the interest shown by healthcare professionals. Patient adherence requires individualized prescription, regular follow-up, and encouragement [4–6, 11, 12]. Recent surveys have shown a change in the attitude of healthcare professionals towards exercise in which they express belief in the positive effects, while indicating lack of support within the healthcare system [13–15].

Our hypothesis was that the main barriers to an integration of exercise in routine care of patients at all stages of CKD and kidney replacement therapy (KRT) are structural and organizational within the European healthcare systems rather than due to resistance among patients or healthcare professionals.

## MATERIALS AND METHODS

This is a descriptive cross-sectional study using anonymized questionnaires to investigate healthcare professionals’ attitudes towards exercise in patients with CKD or on KRT; how healthcare professionals and patients perceive implementation of exercise in practice; the structural support provided by the healthcare system at the kidney unit and national levels in five Western European countries. Countries represented in the European Association of Rehabilitation in Chronic Kidney Disease (EURORECKD) were recruited. Recruitment started in 2019 and ended in 2023. The recruitment period was prolonged due to the COVID-19 pandemic. Of the eight countries invited, Germany, Greece, Italy, Spain, and Sweden participated.

### Terminology

The term kidney unit describes kidney care given in hospitals or in freestanding dialysis facilities. The term ‘physiotherapist’ includes exercise physiologists and similar trained exercise professionals. Exercise is a subcategory of physical activity and is planned, structured, and repetitive with the aim of improving physical performance.

### Randomization process

To ensure broad geographic coverage while maintaining feasibility, we aimed to include approximately one kidney unit per 4 million inhabitants in each participating country. Using available national registries or listings, a random selection process was conducted (by G.T.) to identify eligible kidney units. Where comprehensive national lists were unavailable—as in Germany—random sampling was performed using geographic stratification by zip code to approximate a representative distribution across regions. The number of selected units per country varied based on population size and logistical considerations. Specifically, two centres were selected and participated in both Greece and Sweden. In Germany, 20 centres were selected to reflect its large population and geographical diversity; of these, 13 agreed to participate. In Italy, 13 units were selected and nine participated. In Spain, 10 were selected and two participated. All physicians, nurses, and patients at the selected kidney units were invited to participate in the study.

### Construction and validation of the questionnaires

Five questionnaires were constructed in three steps: first, national experts were involved; second current literature and pre-existing questionnaires were studied; third, questionnaires were constructed targeting organization of and resources for exercise programmes at (i) country level, (ii) kidney unit level, (iii) demographic data of attitudes towards exercise and prescription practices in physicians, (iv) demographic data of attitudes towards exercise in nurses, and (v) demographic data and questions on prescription of exercise in patients.

The questionnaires were validated in two steps: questionnaires 1, 2, and 5 by the national leader using specially constructed validation questions and then by the head of each kidney unit (questionnaire 2), the physicians at the kidney unit (questionnaire 3), and the head nurse(s) (questionnaire 4).

Finally, questionnaires 2–5 were translated into the native language.

### Statistics

The data were analysed at CNR-IFC Clinical Epidemiology and Pathophysiology of Renal Diseases and Hypertension Unit for statistical evaluation. Data are expressed as mean and standard deviation, median and interquartile range, or as absolute numbers and percentages, as appropriate. The 95% confidence intervals have been calculated for all key variables. When possible, heterogeneity among countries for the key questions have been calculated by *I*^2^, which measures heterogeneity by estimating the proportion of variation in prevalence estimates for a given condition that stems from real differences between studies rather than from random chance.

Data analyses were carried out by IBM SPSS Statistics for Windows, Version 27.0. Armonk, NY, USA: IBM Corp. IBM Corp. Released 2020.

### Ethics approval

The study was approved by the Ethics Committee: Lund Dnr: 2018/762; Thessaloniki prot. no. 88 880/2018; Tuscany prot. no 19 856; Bavaria BLAEK no. 2020–1034. In Spain, the approval of the principal investigator’s Ethics committee, Sweden, was accepted. The study was registered at ClinicalTrials.gov Identifier NCT03923972. All participants received written and oral information, and gave written consent.

## RESULTS

### Nurses and physicians

In total, 352 (male 35%) nurses and 143 (male 54%) physicians participated. Both the nurses and physicians had on average a normal BMI, >80% were non- or ex-smokers except for Greek physicians of whom 80% were current smokers. Of the nurses, 61% (CI 55%–94%) were physically active for a median of 180 (120–240) minutes per week as were 58% (CI 50%–66%) of the physicians for a median of 120 (90–180) minutes per week (see Tables 1 and 2).

**Table 1:** Some descriptive data for healthcare professionals by country.

	All countries		Germany		Greece		Italy		Spain		Sweden	
	Nurses	Physicians	Nurses	Physicians	Nurses	Physicians	Nurses	Physicians	Nurses	Physicians	Nurses	Physicians
*N*	352	143	102	44	7	5	198	84	20	6	25	7
Sex, M/F, %	35/65	54/46	11/89	59/41	0/100	60/40	56/44	48/52	20/80	33/67	0/100	57/43
Age years	45 ± 11	46 ± 12	43 ± 14	51.4 ± 9	33 ± 8	46 ± 15	47 ± 9	44 ± 12	44 ± 13	44 ± 13	44 ± 11	46 ± 13
Weight kg	65 [58–75]	66 [59–80]	65 [60–75]	76 [64–84]	65 [60–70]	80 [63–95]	65 [57–75]	63 [57–75]	61 [56–72]	60.5 [54–97]	64 [56–70]	73 [63–78]
Height cm	167 ± 10	171 ± 9	168 ± 8	177 ± 9	169 ± 7.3	173 ± 8	167 ± 11	169 ± 8	165 ± 10	172 ± 12	164 ± 6	175 ± 9
BMI	24 ± 4	24 ± 4	24 ± 4	24 ± 3	23 ± 2.8	26 ± 4	24 ± 4	23 ± 4	25 ± 4	23 ± 4	24 ± 3	23 ± 2
Smoker: non-/ex-/current, %	60/21/19	76/10/14	79/6/15	84/7/5	43/14/43	0/20/80	49/28.8/21.2	75/10/15	45/30/25	67/33/0	84/16/0	86/0/14
Physical exercise, %	61	58	78	79	57	40	48	45	70	50	92	100
Duration, min/week	180 [120–240]	120 [90–180]	152 [100–240]	120 [90–180]	80 [65–110]	105 [90–120]	180 [120–240]	120 [90–180]	140 [120–218]	180 [90–540]	240 [180–285]	200 [142–300]

**Table 2:** Data from all countries. Healthcare professionals’ own level of physical activity, importance they attach to non-pharmacological treatment, and specific physical activity for patients and proportion of patients who have received an exercise training programme.

			Patients				
	Nurses	Physicians	CKD 4–5	PD	HHD	IHD	KT
I engage in regular exercise, %	61 (55–94)	58 (50–66)					
Importance of info to patients on regular exercise, %^a^	96 (94–98)	98 (94–100)					
Importance of exercise as integrated therapy for patients, %^b^							
Nurses	88 (85–89)		91 (87–94)	83 (79–87)	NA	83 (78–87)	90 (86–93)
Physicians		93 (91–95)	94 (89–97)	95 (90–98)	NA	89 (83–94)	94 (89–98)
I have received an exercise programme, %			14 (8–21)	37 (22–54)	14 (4–32)	11 (7–12)	33 (24–44)
Importance of info to patients on nonsmoking, %^a^	96 (94–98)	98 (94–99)					
Importance of info to patients on alcohol consumption, %^a^	95 (93–97)	98 (94–99)					
Importance of info to patients on healthy diet, %^a^	99 (97–100)	99 (95–100)					

^a^Very important or important on a scale from very important—important—uncertain—not important.

^b^7–10 on a Likert scale from 10 to 0 (10 = very important, 0 = not important).

In all countries, 96% (CI 94%–98%) of the nurses and 98% (CI 94%–100%) of the physicians believed it was important to inform patients about the benefits of regular exercise, the positive effects on health of not smoking, moderate alcohol consumption, and a healthy diet. Nurses and physicians, respectively, reported a high rating of importance regarding integrating exercise in routine treatment for patients with CKD 4–5 (91%; 94%), on peritoneal dialysis (PD) (83%; 67%), on institutional haemodialysis (IHD) (83%; 89%), and with a kidney transplant (KT) (90%; 94%) (see Table 2).

### Prescription of exercise in practice by physicians

Of the physicians, 59% (CI 50%–67%) prescribed exercise ‘always or often’ (*I*^2^ 91%), ranging from 15% (CI 6%–29%) of the German physicians to 86% (CI 42%–99%) of the Swedes. *I*^2^ shows how much of the variation in results reflects true differences in the underlying prevalence, as opposed to random sampling error within an individual study.

**Figure 1:**
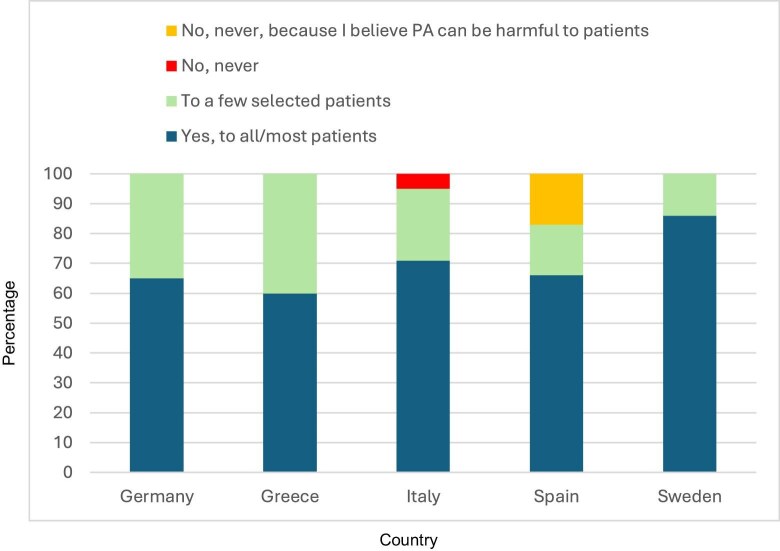
Prevalence by country of the responses to the question ‘Do you regularly advise your patients to increase their level of exercise?’

Six% (CI 3%–12%) ‘rarely’ inquired about patients’ level of exercise, ranging from 0% in Sweden to 100% in Greece (*I*^2^ 99%). Another 69% (CI 61%–77%) regularly advised ‘all or most’ patients to increase their level of exercise (*I*^2^ 0) (see Fig. 1). However, concerning assessment of patients’ level of physical performance, 24% to 26% of the physicians considered it to be ‘important’ for all treatment modalities (*I*^2^ 0), in Greece the facility comprised patients on IHD only. In all countries, 41% (CI 32%–49%) informally assessed their patients’ level of physical performance themselves or with a physiotherapist (*I*^2^ 88%). 25% (CI 19%–31%) believed that patients should have a cardiological, neurological, and physiotherapeutical evaluation before enrolling in an exercise programme, ranging from 13% in Germany to 83% in Spain (*I*^2^ 90%). 49% (CI 42%–56%) believed home-based exercise was most suitable for patients on IHD (*I*^2^ 85%) and 21% (CI 15%–26%) intradialytic exercise (*I*^2^ 78%). Regarding intradialytic exercise 51% (CI 42%–59%) believe that ‘benefits outweigh risks’ (*I*^2^ 63%), and 24% (CI 17%–32%) had ‘no opinion’ (*I*^2^ 86%) (see Table 3).

**Table 3:** Physicians practice and opinions concerning exercise and assessing physical performance by country.

Question	Response	All countries, %	Germany, %	Greece, %	Italy, %	Spain, %	Sweden, %	*I* ^2^ (%)
Do you refer to a specialist, or prescribe directly, and remind your patients to engage in exercise?	Always/Often	59 (50–67)	15 (6–29)	40 (5–85)	78 (68–87)	66 (22–95)	86 (42–99)	91.1
	Sometimes	22 (15–29)	29 (16–45)	60 (15–95)	18 (10–28)	17 (0.4–64)	0	30.8
	Rarely	8 (4–14)	17 (7–32)	0	4 (0.8–10)	17 (0.4–64)	14 (0.4–58)	43.3
	Never	11 (7–18)	39 (24–55)	0	0	0	0	91
	Never, I believe exercise is harmful for patients	0	0	0	0	0	0	NA
Do you ask your patients about their level of exercise?	Always/Often	73 (65–80)	81 (66–91)	0	71 (60–84)	67 (22–95)	43 (9–81)	24.1
	Sometimes	21 (15–29)	17 (7–31)	0	25 (16–36)	33 (4–77)	57 (18–90)	12.9
	Rarely	6 (3–12)	2 (0.1–13)	100 (48–100)	4 (0.8–10)	0	0	99.4
	Never	0	0	0	0	0	0	NA
Do you regularly advise your patients to increase their level of exercise?	Yes, to all/most patients	69 (61–77)	65 (48–78)	60 (15–95)	71 (60–81)	66 (22–95)	86 (42–99)	0
	To a few selected patients	27 (20–35)	35 (22–52)	40 (5–85)	24 (15–35)	17 (0.4–64)	14 (0.4–57)	0
	No, never	3 (0.8–7)	0	0	5 (1–12)	0	0	NA
	No, never, because I believe PA can be harmful to patients	1 (0.02–4)	0	0	0	17 (0.4–64)	0	NA
Do you consider assessing level of physical performance important?	Yes, in CKD 4–5, no KRT	26 (22–31)	25 (17–33)	NA	28 (22–33)	34 (13–59)	24 (9–45)	0
	Yes, HD	24 (20–28)	25 (17–33)	100 (48–100)	22 (17–27)	22 (6–47)	24 (9–45)	96.1
	Yes, PD	25 (21–29)	25 (17–33)	NA	25 (20–31)	22 (6–47)	24 (9–45)	0
	Yes, KT	24 (20–28)	21 (15–30)	NA	25 (20–31)	22 (6–47)	28 (12–49)	0
	No	1 (0.4–3)	4 (1–9)	NA	0	0	0	NA
Do you or an exercise professional objectively assess level of physical performance?	Yes, informally	41 (32–49)	10 (3–24)		60 (48–70)	33 (4–77)	20 (0.5–71)	87.8
	Yes, routinely with questionnaires	2 (0.5–6)	5 (0.6–17)	40 (5–85)	1 (0.3–6)	67 (22–95)	20 (0.5–71)	0
	Yes, routinely with tests	2 (0.5–6)	5 (0.6–17)	20 (0.5–71)	1 (0.3–6)		60 (15–94)	0
	Yes, only in selected patients with questionnaires	3 (0.8–7)	3 (0.1–13)	40 (5–85)	4 (0.8–10)			0
	Yes, only in selected patients, with tests	6 (3–11)	3 (0.1–13)		34 (24–45)			0
	No, never	46 (37–54)	74 (58–87)		1 (0.3–6)			72.1
Which assessments do you consider necessary before enrolling a patient in an exercise programme?	Cardiological evaluation	42 (35–50)	53 (39–67)	40 (5–85)	41 (32–51)		14 (0.4–58)	34.8
	Neurological evaluation	3 (1–7)	2 (0.1–10)	60 (15–95)	5 (2–11)	17 (0.4–64)	43 (10–81)	0
	Physiotherapeutic evaluation	26 (19–31)	23 (13–37)		27 (19–35)	83 (36–99)	29 (4–70)	0
	All of the above	25 (19–31)	13 (6–26)		26 (18–34)		14 (0.4–57)	90.5
	None of the above	4 (1–7)	9 (3–21)		1 (0.2–4)			50.9
Which type of exercise do you consider most suitable for patients on HD?	Intradialytic exercise	21 (15–26)	31 (22–41)	40 (5–85)	5 (1–12)	33.3 (4–77)	27 (6–61)	77.7
	Home-based programmes	49 (42–56)	34 (25–44)	60 (15–94)	72 (60–80)	33.3 (4–77)	55 (23–83)	84.8
	Assisted in-centre	28 (21–34)	35 (27–46)		21 (13–31)	33.3 (4–77)	18 (2–51)	45.9
	None of the above	2 (1–5)			2 (0.8–8)			87.8
What is your opinion regarding intradialytic exercise	Risks outweigh benefits	11 (6–17)	5 (0.6–16)		14 (7–23)	40 (5–85)		52.4
	Benefits outweigh risks	51 (42–59)	78 (62–89)	60 (15–94)	37 (20–57)	60 (15–94)	43 (10–82)	62.9
	Benefits and risks are equal	14 (8–20)	10 (3–23)	40 (5–85)	15 (8–25)		14 (0.4–57)	0
	No opinion	24 (17–32)	7 (2–20)		34 (24–45)		43 (10–82)	86.2

### Healthcare structure’s provision of resources for exercise for kidney patients

**Figure 2:**
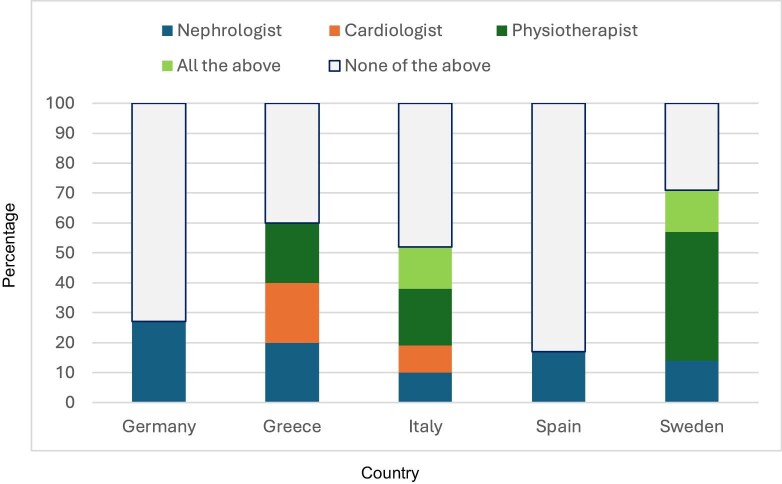
Prevalence by country of the responses to the question ‘Which specialist is primarily responsible at your hospital/facility for prescribing exercise outside the dialysis unit for patients on dialysis?’

Forty-eight% (CI 39%–56%) did not have a physiotherapy unit at their hospital/facility (*I*^2^ 98%), 41% (CI 33%–49%) had one but not dedicated to kidney patients (*I*^2^ 92%): in Italy 70% (CI 58%–79%) and Sweden 14% (CI 0.4%–57%) did not (*I*^2^ 92%). Six% (CI 3%–12%) had a physiotherapy unit for kidney patients: Italy had one% (CI 0.03%–7%) and Sweden had 86% (CI 42%–99%) (*I*^2^ 97%). Seventy% (CI 62%–78%) did not have dedicated staff for prescribing and supervising exercise programmes for kidney patients (*I*^2^ 94%), seven% (CI 3%–12%) had physiotherapists: Italy had four% (CI 1%–10%) and Sweden had 71% (CI 29%–96%) (*I*^2^ 87%). Of all kidney units, 85% (CI 78%–90%) did not have dedicated exercise programmes for any of the treatment modalities (*I*^2^ 0), six% (CI 3%–12%) had them for patients on dialysis only (*I*^2^ 98%), and eight% (CI 4%–13%) had them for all treatment modalities (*I*^2^ 97%). Two countries had dedicated exercise programmes for all treatment modalities: Italy had five% (CI 1%–12%) and Sweden had 86% (CI 42%–99%) (*I*^2^ 97%). Fifty-five% (CI 46%–64%) did not have a dedicated specialist outside the dialysis unit for prescribing exercise to patients on dialysis (*I*^2^ 70%, see Fig. 2 and Table 4).

**Table 4:** Structure at hospital/facility and kidney unit regarding availability of physical activity and exercise.

Question	Response	All countries, %	Germany, %	Greece, %	Italy, %	Spain, %	Sweden, %	*I* ^2^ (%)
Is there a physiotherapy unit at your hospital/facility?	Yes, but not with exercise programmes for kidney patients	41 (33–49)			70 (58–79)		14 (0.4–57)	92.4
	Yes, it is involved with exercise programmes for kidney patients	6 (3–12)			1 (0.03–7)		86 (42–99)	97
	Yes, for patients with neuro-muscular disease	5 (2–10)			8 (4–17)			NA
	No, there is no physiotherapy unit	48 (39–56)	100 (91–100)	100 (48–100)	21 (13–31)	100 (54–100)		97.8
Does your kidney unit have dedicated exercise programmes for patients?	On dialysis	6.3 (3–12)	7 (2–20)	100 (48–100)	9 (3–17)		14 (0.4–57)	97.6
	CKD no KRT	0.7 (0.02–4)	2 (0.6–13)		1 (0.03–7)			0
	KT	0.7 (0.02–4)			1 (0.03–7)			NA
	All the above	7.7 (4–13)			5 (1–12)			97
	No, none	84.6 (78–90)	91 (77–97)		84 (75–91)	100 (54–100)	86 (42–99)	0
Does your hospital/facility have dedicated staff for prescribing and supervising exercise programmes for kidney patients?	Yes, staff from the Nephrology Dept	2 (0.4–6)	3 (0.1–13)	40 (5–85)				68.8
	Yes, physiotherapists	7 (3–12)	5 (1–17)	20 (0.5–71)	4 (1–10)		71 (29–96)	86.8
	Yes, graduates in exercise science	3 (1–7)			2 (0.3–8)			0
	Yes, nurses	18 (12–26)	63 (47–78)					NA
	No, none of the above	70 (62–78)	29 (16–45)	40 (5–85)	94 (86–97)	100 (54–100)	29 (4–71)	93.7
Which specialist is primarily responsible at your hospital/facility for prescribing physical exercise outside the dialysis unit for patients on dialysis?	Nephrologist	16 (10–23)	27 (15–44)	20 (0.5–72)	10 (4–18)	17 (0.4–64)	14 (0.4–58)	NC
	Cardiologist	6 (3–11)		20 (0.5–72)	9 (3–17)			0
	Physiotherapist	14 (9–21)		20 (0.5–72)	19 (11–30)		43 (10–82)	0
	All the above	9 (5–15)			14 (7–24)		14 (0.4–58)	0
	None of the above	55 (46–64)	73 (56–85)	40 (5–85)	48 (36–59)	83 (36–99)	29 (4–71)	70.3

NC, not calculable.

### Healthcare structure’s reimbursement for costs of physiotherapists

In Germany, health insurance is compulsory, provided either through statutory health insurance or substitutive private health insurance. Compliance with regulations is mandatory, otherwise the prescribing doctor is personally liable for recourse payments. The major barriers in Germany are unclear reimbursement policies for exercise in CKD, along with the considerable risk of personal financial liability for the prescribing physician. On the basis of the results from the DiaTT study, a change of this ruling is currently being discussed [6].

Greece, Italy, and Spain have national healthcare systems. The integration of exercise rehabilitation programmes in CKD management is limited as it is not supported by the national healthcare reimbursement systems. There are no standardized national protocols that formally put exercise interventions and the role of the physiotherapist into chronic disease care pathways, resulting in fragmented implementation and limited referral by physicians.

Sweden has county level healthcare services, which is available to all residents; patients pay a fixed maximum contribution per year for healthcare and treatment by a physiotherapist is included. It is up to each hospital and kidney unit to allot resources to a physiotherapist within the budget provided. The National Board of Social Affairs and Health recommends physical activity for people with chronic diseases and has implemented ‘Physical Activity on Prescription’, which enables healthcare professionals to prescribe subsidized physical activity that is included in the budget for pharmaceuticals. There is also an official pharmacopoeia for prescription of physical activity for most chronic diseases.

### Patients

A total of 1235 patients participated from all countries, comprising 137 patients (male 62%) with CKD 4–5, 40 patients (male 60%) on PD, 30 (male 63%) patients on home haemodialysis (HHD), 928 (male 59%) patients on IHD, and 100 (male 62%) patients with KT. The average age ranged from 58 ± 12 years for patients with KT to 75 ± 9 years for patients on HHD. Most patients had no walking limitations (CKD 4–5 71%; PD 83%, HHD 50%, IHD 64%, KT 86%), 75% to 90% reported informal support from a family member, and 14% to 49% had formal care from a professional caregiver (see Table 5).

**Table 5:** Some descriptive data for patients in all countries on different treatment modalities.

	CKD 4–5	PD	HHD	IHD	KT
Total Number of patients	137	40	30	928	100
Germany	43	13	26	293	25
Greece	0	0	0	49	0
Italy	27	20	3	411	19
Spain	16	0	0	144	2
Sweden	51	7	1	31	54
Sex, M/F, %	85/52	60/40	63/37	59/41	62/38
Age, years	68 ± 15	62 ± 19	75 ± 9	68 ± 14	58 ± 13
Marital status, %					
Married/cohabiting	61	75	57	60	68
Divorced/separated	7	5	3	8	10
Widowed	17	2	20	16	6
Never married/cohab	15	18	20	16	16
Highest educational level, %					
None	5	0	6.7	4	2
Elementary school	42	10	23.3	29	22
Incomplete high school	10	13	6.7	11	16
High school	16	28	0	23	30
Vocational training	15	20	43.2	15	10
University degree	7	21	6.7	12	17
Postgrad	3	5	6.7	3	3
Other	2	3	6.7	3	0
Distance to renal unit, km	12|3–25]	19[8–31]	10[3–19.5]	8 [3–6]	13[4–30]
Travel time to renal unit, min	25[15–35]	25[15–40]	17.5[10–25]	20 [10–30]	20[15–35]
Mobility, %					
No walking limitations	71	83	50	64	86
Walk with a cane or human support	25	10	37	27	11
Wheelchair	4	7	13	8	3
Bedridden	0	0	0	1	0
Informal support from family member, %	75	90	83	83	83
Formal care by professional, %	25	49	30	48	14

### Patients’ perceptions of prescription of exercise in practice

**Figure 3:**
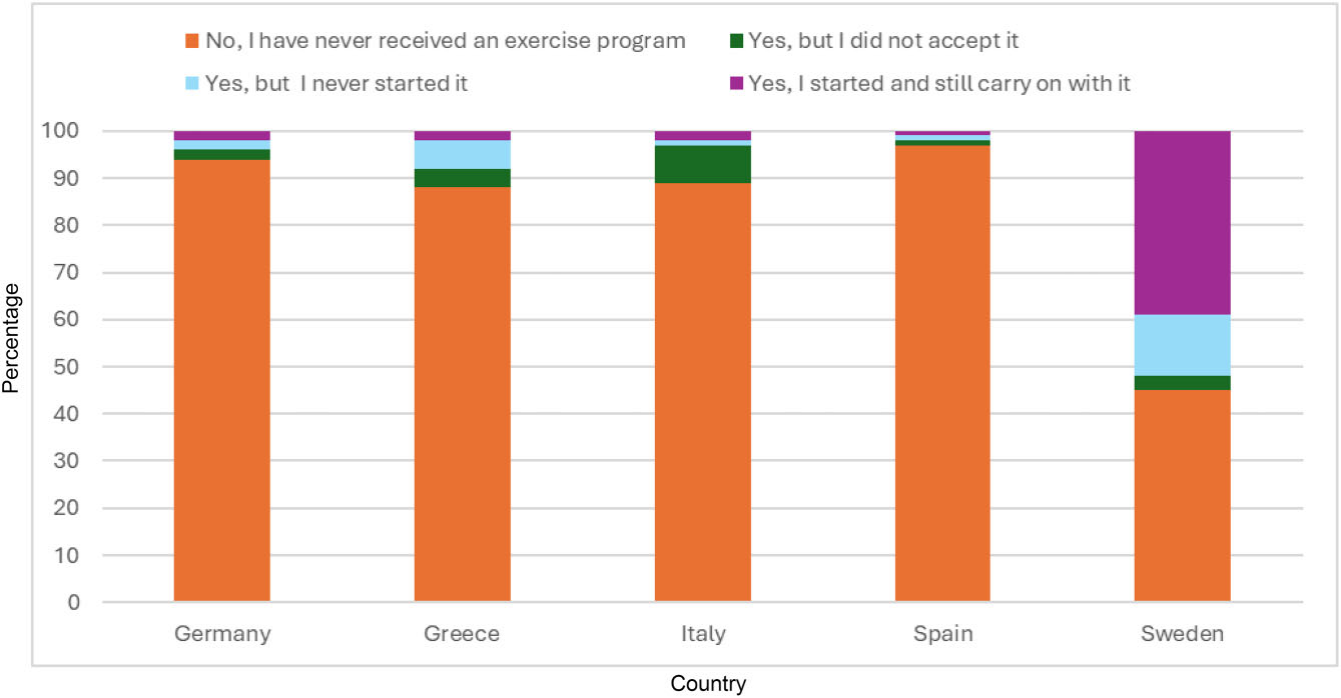
Prevalence by country of the responses of patients on haemodialysis to the question ‘Have you ever received a rehabilitation/exercise programme from a staff member at your kidney unit?’

Of patients with CKD 4–5, 14% reported having received an exercise programme, 37% of patients on PD, 14% on HHD, 11% on IHD and 33% with KT. 9% (CKD), 16% (PD), 7% (HHD), 3% (IHD), and 11% (KT) of all patients in each treatment modality had continued with the exercise programme, constituting a self-reported adherence between 33% and 64%. See Table 6, see Fig. 3 for prevalence in patients on IHD by country.

**Table 6:** Patients’ responses to the question ‘Have you ever received a rehabilitation/exercise programme from a staff member at your nephrology or dialysis centre?’

		All countries, %	Germany, %	Greece, %	Italy, %	Spain, %	Sweden, %
CKD	No, I have never received an exercise programme	86	91	NA	96	100	74
	Yes, but I did not accept it			NA			
	Yes, I never started it	5		NA			12
	Yes, I started and I still carry on with it	9	9	NA	4		14
PD	No, I have never received an exercise programme	63	91	NA	55	NA	43
	Yes, but I did not accept it	16	9	NA	25	NA	
	Yes, I never started it	5		NA	5	NA	14
	Yes, I started and I still carry on with it	16		NA	15	NA	43
HHD	No, I have never received an exercise programme	85.7	88	NA	100	NA	NA
	Yes, but I did not accept it	3.6	4	NA		NA	NA
	Yes, I never started it	3.6	4	NA		NA	NA
	Yes, I started and I still carry on with it	7.1	4	NA		NA	NA
IHD	No, I have never received an exercise programme	91	94	88	89	97	45
	Yes, but I did not accept it	4	2	4	8	1	3
	Yes, I never started it	2	2	6	1	1	13
	Yes, I started and I still carry on with it	3	2	2	2	1	39
KT	No, I have never received an exercise programme	66.4	87	NA	32	100	69
	Yes, but I did not accept it	12.2	4	NA	58		
	Yes, I never started it	10.2		NA			15
	Yes, I started and I still carry on with it	11.2	9	NA	10		16

NA, not applicable

## DISCUSSION

In this European survey of attitudes towards prescription and implementation of and allocated resources for exercise in patients with CKD 4–5 and on KRT, we found that >95% of the nurses and physicians believed in the benefits of exercise, ∼70% regularly asked patients about their level of exercise and regularly advised them to increase their level of exercise. However, only 25% considered it important to assess patients’ physical performance and <60% prescribed exercise regularly, comprising 15% of the physicians in Germany compared with 78% in Italy and 86% in Sweden. Most patients replied that they had never received an exercise programme, ranging from 63% of patients with PD to 91% on IHD. Of the patients who had received an exercise programme between one- and two-thirds of all patients adhered to it. The patients’ answers reflect the healthcare structure in the respective countries, given that most units did not have physiotherapists who could assess patients’ physical performance, prescribe, and follow progress.

Sweden was the only country with dedicated physiotherapists for patients on IHD and the only country with a reimbursement structure covering the cost of physiotherapists for all stages of CKD and KRT. In no other country studied was reimbursement indisputable for patients with CKD or KRT. In Germany, physicians risk receiving a recourse claim from the health insurance company should they prescribe exercise to patients with CKD or on KRT, for which they are personally liable. In Greece, Italy, and Spain exercise for patients with CKD or on KRT is not reimbursed unless complicated by other comorbidities included in the reimbursement regulations.

There are marked differences in healthcare in the participating countries. Germany has compulsory health insurance; Greece, Italy, and Spain have a national health system; and Sweden has a county health system. The bottom line for each of these systems is that, except for Sweden, there is no reimbursement policy in place to cover the costs of physiotherapists for patients with CKD or on KRT. Moreover, none of the countries, except Sweden, has a national protocol explicitly advocating exercise as an integral part of comprehensive kidney care.

Obviously, the reimbursement system governs what type of healthcare is given, irrespective of the attitudes of the healthcare professionals or patients’ needs and wishes.

This survey found that nurses and physicians strongly believed in the benefits of exercise for all patients irrespective of treatment modality, indicating a profound shift of attitude. Most physicians inquired about their patients’ level of exercise and prescribed exercise. Earlier surveys show that physicians were sceptical of the feasibility of exercise for their patients and were wary of prescribing it as they did not perceive that patients would be interested or have the energy to exercise, nor did they feel convinced of the benefits nor feel confident prescribing exercise [7–9, 16].

During the last decade there have been some important large long-term RCTs in patients on dialysis, unequivocally showing the benefits of exercise on physical performance, hospitalization, and mortality [4, 6], which seem to have affected the attitudes of healthcare professionals, but not reimbursement policies of healthcare providers [7–9].

Few units in this study assessed physical performance or engaged patients in structured exercise. In fact, 85% of all kidney units did not have dedicated exercise programmes for their patients, which is corroborated by most of the patients reporting that they never had received an exercise programme. About 6% of all units had physiotherapists and a physiotherapy unit at their disposal. In Sweden both units studied provided dedicated physiotherapy and leaflets on the benefits of exercise to their patients.

This study showed a marked discrepancy between the importance healthcare professionals attach to exercise and resources allocated. There was also a discrepancy between the strong belief held that exercise is beneficial, which caused physicians to recommend exercise to their patients, and the value attached to assessing each patient’s level of physical performance. Neither the physicians nor the nurses have the training or the time, nor are these tasks part of their job descriptions. Physiotherapists are dedicated professionals trained to assess physical performance and to prescribe exercise programmes to address each person’s weaknesses, physical needs, and goals. Only a very small proportion of patients had the benefit of meeting a physiotherapist.

Several large RCTs with self-administered exercise at home or at a gym have shown that patients on dialysis and with CKD 3–5 have a high rate of adherence over a period of 6 to 12 months [4, 5, 17]. Another large RCT of 12 months’ duration showed that intradialytic exercise was feasible with a high rate of adherence [6]. In these studies, a crucial success factor was regular assessment of physical performance with adjustment of the exercise dose, personal support, and encouragement provided on a regular basis by nurses in the EXCITE trial and by physiotherapists in the RENEXC and DiaTT trials.

Although physicians in this survey are strong advocates of the benefits of exercise for kidney patients, they prescribe or more probably recommend exercise without prior assessment of the patient’s physical performance and without assessing the effects of the therapy. This discrepancy is unsurprising given the lack of physiotherapists to whom patients could be referred. A pharmacological agent would not be prescribed without an assessment of the patient’s baseline status, knowledge of the medication, a definition of the aims hoped to be achieved, and regular follow-up. It is necessary that exercise, a non-pharmacological therapy with evidence-based effects, is managed accordingly.

This European survey clearly shows that the lack of physiotherapeutic expertise and presence at the kidney units is a structural problem within the healthcare systems in the countries studied. In most countries, exercise is not recognized by healthcare providers as an integral part of the care of kidney patients and funding is not provided. These findings are confirmed by recent studies reporting that lack of funding and physiotherapy resources are important barriers [14, 15, 18–20]. Sweden was the exception: both kidney units studied had a dedicated physiotherapist, one unit had a gym adjacent to the dialysis unit and the other unit assessed all patients on KRT biannually and prescribed home- or gym-based exercise. Patients with CKD 4–5 were referred to a physiotherapist in primary care.

This European study substantiates previous findings and highlights the importance of convincing policy makers and healthcare providers of the benefits of exercise in kidney patients. Moreover, it points to a strategy that has shown to be successful in Sweden, with regular assessment and prescription of exercise by dedicated physiotherapists integrated in routine care.

However, if healthcare systems are going to be willing to provide resources, they will want to be convinced that the benefits can be translated into cost reductions. Several trials in patients on dialysis have shown that exercise reduces the number of hospitalizations [4, 6] and other concomitant costs [16]. Several long-term large RCTs in patients with CKD 3–5 and on dialysis have unequivocally shown that all aspects of physical performance improve, which implicitly enhances the patient’s ability to care for themselves and remain mobile making them less dependent on both informal and professional carers [3–6]. To summarize, these benefits translate into less money needing to be spent on in-hospital care and professional caregiving at home or in a care-home.

This study has some limitations. While random selection aimed to reduce selection bias, differential participation rates across countries may have limited full representativeness and introduced some degree of selection bias. Given the pragmatic nature of the study, we did not explore potential for internal bias and heterogeneity of attitudes. The spectrum of views among the healthcare professionals is not fully explored in the current analysis. Future research should investigate these internal differences through in-depth qualitative interviews.

The focus in the present study was healthcare professionals’ attitudes, and the structure of healthcare, patients’ attitudes, and perception of barriers are lacking and will be investigated in a further study. Only Western European countries are represented. Some strengths are the number of healthcare professionals and patients included; that it comprises countries from the north, south, and middle of Western Europe, large and small countries, and countries with different healthcare systems; and that all treatment modalities are included.

In conclusion, healthcare professionals regard exercise as an important element of care in kidney patients, although, they do not assess patients’ physical performance nor provide structured exercise programmes, mainly due to lack of physiotherapy resources and reimbursement strategies within the healthcare system. Sweden is the exception. Thus, it is high time that healthcare systems recognize the beneficial effects of exercise and provide adequate resources. This would improve health and wellbeing for all patients with CKD and on KRT.

## Data Availability

Data are available on request from Clinical Epidemiology and Pathophysiology of Renal Diseases and Hypertension Unit.

## Notes

The EUSUREX Working Group: Germany: David Plate, DaVita-Nierenzentrum, Aachen; Horst Berger, Dialysepraxis, Bad Salzungen; Andreas Fürsch, KfH-Nierenzentrum, Bad Soden; Thomas Döltz, KfH-Nierenzentrum, Bamberg; Norgit Meyer, KfH-Nierenzentrum, Bautzen; Cosima Pohle, KfH-Nierenzentrum, Berlin-Charlottenburg; Dominik Kahle, Diaverum-Nierenzentrum Hamburg-Wandsbek; Michael Schömig, NEPHRO-Dialyse, Heilbronn; Pawlos Ichtiaris, Nephrologisches Zentrum, Marktredwitz; Roman Günthner, PHV-Dialyse, Melsungen; Gunnar Bücker, Dialyse Praxis, Osnabrück; Bernadette Assenbrunner, KfH-Nierenzentrum, Pfaffenhofen; Kerstin Fiegler, Dialysezentrum, Rüdersdorf. Greece: Vasiliki Michou; University of Western Macedonia, Kozani; Ourania Kougioumtzidou, Alpha Nefrodinamiki Unit, Trilofo; Zagorianakos Antonios, Renal Unit, BioClinic, Athens, Greece; Kakaletri Konstantina, Protypo Dialysis Center of Galatsi, Athens; Liakopoulos Vasileios, 2nd Department of Nephrology, AHEPA Hospital, Thessaloniki. Italy: Maurizio Gallieni, Milan University Hospita; Manuel Ferraro, Gemelli University Hospital, Rome; Elena Mancini, Malpighi Hospital, Bologna; Stefano Santarelli, Jesi Hospital, Jesi, Italy; Antonio Selvi, ASL, Umbri; Paolo Monardo, Papardo Hospital, Messina; Giovanni Campolongo, Napoli; Donatella Andreoli, Acri Hospital, Acri; Annalisa Carta, S. Francesco Hospital, Nuoro. Spain: Shaira Martínez Vaquera, Medical Director of Diaverum, Madrid. Sweden: Karl Bjurström, Halland’s Hospital, Halmstad; Lars Ekholm and Noomi Lundgren, Ljungby Hospital, Ljungby.
